# Cost and cost-effectiveness analysis of treatment for child undernutrition in low- and middle-income countries: A systematic review

**DOI:** 10.12688/wellcomeopenres.15781.2

**Published:** 2020-10-05

**Authors:** Rebecca G Njuguna, James A Berkley, Julie Jemutai

**Affiliations:** 1 KEMRI-Wellcome Trust Research Programme, Kilifi, Kenya; 2 Department of Public Health, School of Health and Human Sciences, Pwani University, Kilifi, Kenya; 3 The Childhood Acute Illness & Nutrition (CHAIN) Network, Nairobi, Kenya; 4 Centre for Tropical Medicine and Global Health, Nuffield Department of Clinical Medicine, University of Oxford, Oxford, UK

**Keywords:** Economic burden, cost, cost effectiveness analysis, undernutrition, malnutrition, community-based, low and middle-income countries

## Abstract

**Background**: Undernutrition remains highly prevalent in low- and middle-income countries, with sub-Saharan Africa and Southern Asia accounting for majority of the cases. Apart from the health and human capacity impacts on children affected by malnutrition, there are significant economic impacts to households and service providers. The aim of this study was to determine the current state of knowledge on costs and cost-effectiveness of child undernutrition treatment to households, health providers, organizations and governments in low and middle-income countries (LMICs).

**Methods:**  We conducted a systematic review of peer-reviewed studies in LMICs up to September 2019. We searched online databases including PubMed-Medline, Embase, Popline, Econlit and Web of Science. We identified additional articles through bibliographic citation searches. Only articles including costs of child undernutrition treatment were included.

**Results: **We identified a total of 6436 articles, and only 50 met the eligibility criteria. Most included studies adopted institutional/program (45%) and health provider (38%) perspectives. The studies varied in the interventions studied and costing methods used with treatment costs reported ranging between US$0.44 and US$1344 per child. The main cost drivers were personnel, therapeutic food and productivity loss. We also assessed the cost effectiveness of community-based management of malnutrition programs (CMAM). Cost per disability adjusted life year (DALY) averted for a CMAM program integrated into existing health services in Malawi was $42. Overall, cost per DALY averted for CMAM ranged between US$26 and US$53, which was much lower than facility-based management (US$1344).

**Conclusion**: There is a need to assess the burden of direct and indirect costs of child undernutrition to households and communities in order to plan, identify cost-effective solutions and address issues of cost that may limit delivery, uptake and effectiveness. Standardized methods and reporting in economic evaluations would facilitate interpretation and provide a means for comparing costs and cost-effectiveness of interventions.

## Introduction

Malnutrition (undernutrition, overweight and micronutrient deficiencies) is a major underlying factor for mortality, morbidity and poor child development ^1, 2^. Undernutrition is associated with lower achievement in education, reduced employment achievement and health status in adulthood and low birthweight in offspring, creating an intergenerational cycle ^2, 3^. Worse effects in children are experienced during their first 1000 days, owing to their higher nutritional requirements and fragile nature ^4, 5^. Only a small fraction of these deficits is reversible during childhood and adolescence, especially if the children remain in impoverished environments ^5, 6^.

Despite efforts by national and international organizations, malnutrition rates remain alarmingly high. Undernutrition is estimated to cause approximately half of all under five deaths, close to 3.1 million deaths annually ^4^. Moderate and severe stunting and wasting affected close to 155 million and 17 million under five children, respectively, by 2016 ^7^. The highest prevalence of wasting is in low- and middle-income countries (LMICs), with sub-Saharan Africa and South Asia accounting for majority of cases ^4^. Poverty, adverse climatic conditions, policies, corruption, social cultural and religious factors are major contributing factors to the high prevalence of child undernutrition in sub-Saharan Africa ^8^.

Until recently, all children suffering from severe acute malnutrition (SAM) were treated as inpatients, which was a major limitation due to inaccessibility of health facilities ^1, 9^. In 2007, the World Health Organization (WHO) endorsed community-based management of acute malnutrition (CMAM) to treat uncomplicated SAM cases and moderate acute malnutrition (MAM) cases in the community ^10^. CMAM constitutes community mobilization, treating uncomplicated SAM and MAM cases as outpatients with ready-to-use therapeutic food (RUTF) and antimicrobials to treat infections ^11^. Cases with medical complications are still recommended to be admitted to inpatient units and are discharged to outpatient care once stabilized and feeding adequately, rather than full nutritional rehabilitation being conducted in the inpatient setting.

### Economic impact

While there is a lot of research ongoing on the health and human impacts of child undernutrition, there is paucity of information on the economic impacts that necessitate further exploration. The long-term effects of undernutrition on the child’s economic potential translate to a reduction in national productivity ^12^. Studies show that children affected by malnutrition in early life risk losing a significant percentage of their lifetime earnings ^13^. For instance, a 1% less attained height is estimated to contribute to a reduction of 2.4% earnings in adulthood ^13^.

Malnutrition is responsible for an 11% yearly Gross National Product (GNP) loss in Africa and Asia ^14^. These economic losses are largely due to provider costs of treating undernutrition and its associated infections, reduced educational performance and lower agricultural activity ^15^. Thus, undernutrition is a major setback towards poverty eradication and attainment of sustainable development goals (SDGs). Support for nutrition interventions is an investment for the future. For instance, attainment of the 40% stunting reduction target by the World Health Assembly by 2025 could result in a cumulative addition Gross Domestic Product (GDP) of US$7 billion in Uganda ^13^.

Costs incurred by households with undernourished children have largely been ignored although such costs may exceed costs to the government ^15, 16^. This is predominantly due to the high expenditure on health care (out-of-pocket costs) during malnutrition treatment and indirect costs, including the opportunity cost of time spent away from normal duties while taking care of the sick children or attending clinics ^15^. To cover these costs, families may borrow or sell assets and be highly dependent on other family members and the community, majorly affecting their economic productivity.

The aim of this systematic review was to determine the current state of knowledge on the costs and cost-effectiveness of child undernutrition treatment(s) to households, health providers, organizations and governments in LMICs. The findings will inform health researchers, policy makers, non-governmental organisations and the private sector to plan, identify cost-effective solutions and address issues of cost to providers and households that may limit delivery, uptake and effectiveness. We only included studies that assessed the cost of treatment interventions (for children with anthropometrically defined wasting or kwashiorkor). Interventions ranging from supplementary feeding for children with moderate acute malnutrition and therapeutic feeding and other treatments for children with severe acute malnutrition, including during community-based management of severe acute malnutrition (CMAM) as well as facility-based outpatient and inpatient treatment. We excluded prevention interventions, screening and treating micronutrient deficiencies as they are broader topics worthy of their own reviews.

## Methods

### Information sources

This systematic review followed the Preferred Reporting Items for Systematic reviews and Meta-Analyses (PRISMA) guidelines ^17^. We conducted a literature search for all studies published in English or French up to September 2019 in the following electronic databases; PubMed-Medline, Embase, Popline, Econlit and Web of Science. We also sought additional published articles through Google Scholar and bibliographic citation searches.

### Inclusion and exclusion criteria

We included articles that (1) were published in English or French; (2) involved treatment interventions for anthropometric undernutrition; (3) had children (below 18 years) as the sample in the study; (4) had cost components or involved economic evaluation and; (5) were conducted in low and middle-income countries.

We excluded articles that did not meet our criteria in two stages. At the initial stage (by title and abstracts) if the study involved an adult population, was done in a high-income country, included overweight/obesity or involved micronutrient deficiencies with no anthropometric undernutrition. At the second stage (full article review) if the article was a study protocol, had reported global cost estimates of child undernutrition treatment or was a review article.

### Search strategy

We used the National Health Service Centre for Reviews and Dissemination ^18^ recommendations to develop a search strategy where the review question was broken down to search terms ( Table 1). We also used Medical Subject Headings (MeSH) terms in addition to the main search terms. We combined the search terms using Boolean operators such as “AND” and “OR” as necessary.

**Table 1.** Search terms as included in the databases.

(cost OR “financial burden” OR “economic burden” OR “financial cost” OR “economic cost” OR expens* OR expend* OR spending) AND (malnutrition OR undernutrition OR undernourish* OR malnourish* OR wasting OR “wasted” OR SAM OR MAM OR “Severe Acute Malnutrition” OR “Moderate Acute Malnutrition” OR kwashiorkor OR “nutritional oedema” OR “nutritional edema") AND (child OR children OR baby OR babies OR infant OR infants)

### Screening of articles

We exported and combined articles retrieved from the different databases in Endnote X8 ^19^ to remove duplicates. We used the Rayyan web app ^20^ for screening of the articles. Two reviewers screened the titles and abstracts independently. We resolved disagreements by consensus. The process was repeated for full article review until relevant articles were selected.

### Data extraction

We collected all relevant information required for analysis using a data extraction template designed in Microsoft Excel 2013. We extracted details on author, year of publication, country, data year, number of children, age range of the children, the study perspective, the time horizon (period between data collection and analysis), type of economic evaluation conducted, analytical approach used, intervention/s studied, comparator/s, cost per DALYs, cost per life years saved, cost per case averted, incremental cost effectiveness ratio (ICER), direct medical costs, direct non-medical costs, indirect costs, total costs, coping strategies and cost drivers.

### Quality assessment of the studies

We assessed the quality of the included studies using the Global Health Cost Consortium (GHCC) guidelines ^21^. The GHCC guidelines consist of 17 items within four main sections designed to evaluate costing studies: 1) study design and scope, 2) service and resource use measurement, 3) valuation and pricing, 4) analyzing and presenting results. Each item was rated by the extent of reporting in the following categories: “1=satisfied” or “0=not satisfied” and “X=not applicable”. For each reviewed study, the “not applicable” rating was acceptable for three items in the GHCC guidelines: “Amortization of capital costs”, “Discounting and inflation” and “use of shadow prices”. This was because amortization of capital costs, discounting and inflation only applies for studies reporting costs over a period of more than one year while use of shadow prices applies for studies valuing inputs without market prices. The total number of articles reporting by each item was then summed up.

### Cost and cost-effectiveness analysis

We classified the extracted cost data into direct medical, direct non-medical and indirect costs. The direct medical costs included expenditure on medication (drugs and diagnostic tests), supplementary feeds (therapeutic food), capital (buildings, equipment and supplies), personnel (staff salaries) and administrative costs (training, monitoring and supervision of activities and consultation fees). Direct non-medical costs included travel, food expenses for caregivers and any other person accompanying them and costs incurred to cover household chores usually done by the families. Indirect costs included the opportunity cost of time the guardians or caregivers spent away from their daily productive routine. We also reviewed data on the cost-effectiveness of CMAM compared to facility based management of malnutrition. We extracted data on cost per DALY gained/averted, cost per life year saved and cost per child treated/recovered from the included studies.

### Statistical analysis

We used R version 3.4.1 ^22^ for all statistical analyses. We converted all costs to US dollars using a currency converter ^23^ for each data year reported. We reported the means, medians and ranges of the direct and indirect costs according to the perspectives adopted by the included studies. The mean and median costs reported were used to assess the main cost drivers for each perspective. We also reviewed coping strategies reported by the included articles. A comprehensive meta-analysis for comparison of costs across the included studies was not done due to hetereogeneity in the costing methods and the interventions assessed.

## Results

### Search results

**Figure 1.**
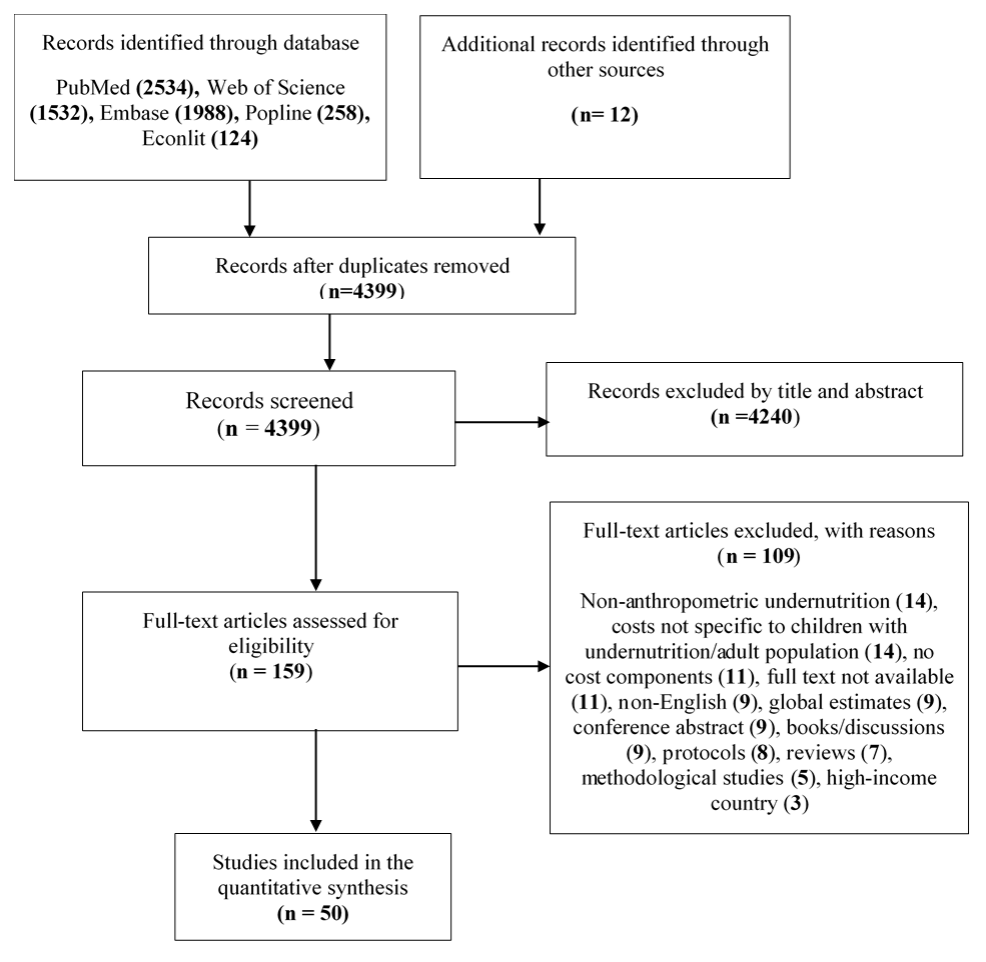
Flowchart showing the search, selection and inclusion of studies.

The literature search yielded 6436 articles: 6424 titles and abstracts through database searching and an additional 12 records through bibliographic citation searches. A total of 4399 articles (excluding duplicates) were selected for title and abstract evaluation. Full-text articles were then obtained for the 159 articles considered potentially eligible for inclusion and full-text articles were obtained; 50 of which met the inclusion criteria ( Table 2). We excluded 109 articles after full article review, mostly with no anthopometric undernutrition or no cost components. Figure 1 shows the flow of selection and inclusion of the studies.

**Table 2.** Characteristics of the included studies in the review.

No	Author	Year	Country	Study design	Type of economic evaluation	Perspective of study	Analytical approach	Intervention	Sample size(n)	Age (months)	Economic Outcome
1	Abdul-Latif *et al.* ^31^	2014	Ghana	Retrospective cross-sectional study	Cost analysis	Societal	Activity- based costing	Community-based management of SAM	40	6 to 59	Cost per child: $805.36
2	Ackatia *et al.* ^32^	2015	Mali	Cluster randomized trial	Cost analysis	Provider	NR	Supplementary feeds (community-based) a) RUSF b) CSB++ c) Locally processed, fortified flour (Misola) d) LMF	a)344; b)349; c)307; d)284;	6 to 35	Cost of supplements;: a) $0.38 for 92g b) $0.22 for 127g c) $0.21 for 125 g d) LMF =$0.18 for 129 g.
3	Akram *et al.* ^33^	2016	Pakistan	Retrospective cohort	Cost analysis	Program	NR	Nutritional rehabilitation (home based-high density diet, parental counselling & monitoring)	123	15.5 ± 8.5	Total cost per child for rehabilitation: $34.31 100g of high density diet cost $0.22
4	Ashworth *et al.* ^34^	1997	Bangladesh	Longitudinal, prospective and controlled trial	Cost- effectiveness	Institutional & parental	Bottom-up approach	a) Inpatient management b) Day care c) Domiciliary	437	12 to 60	a) $159 b) $63.8 c) $38.8
5	Bachmann ^35^	2009	Zambia	Decision analytical modelling	Cost- effectiveness	Healthcare care providers	Modelling approach	Community-based therapeutic care (CTC) vs hypothetical no treatment	2523	<60	Mean cost per child was $203 CTC cost $53 per DALY gained and $ 1760 per life year saved
6	Bai ^36^	1972	India	Prospective cohort	Cost analysis	Hospital and families	NR	Domiciliary management of PEM (special diet)	25	<60	Hospital costs Rs. 525 Parent costs = Rs. 100–150
7	Bagriansky *et al.* ^29^	2014	Cambodia	Model study of economic losses due to malnutrition		Government	Modelling approach		-	-	Economic losses due to; Wasting = $18.8 Underweight = $22.3 Stunting = $128
8	Bredow *et al.* ^37^	1994	Jamaica	Prospective cohort	Cost analysis	Healthcare care providers	NR	Community based approach to treatment of SAM (dietary advice, antibotics, anthelminthics & vitamin supplements)	36	<36	Medication cost US$14 per child for every six months Milk and fat food cost US$2
9	Chapko *et al.* ^38^	1994	Niger	Randomized clinical trial	Cost analysis	Healthcare care providers	Bottom-up approach	Hospital vs ambulatory nutitional rehabilitation	100	5 to 28	a) Hospital= 760 FCFA/patient/day b) Ambulatory = 720 FCFA/patient/ day The mean cost for; a) Hospital rehabilitation = 22881 FCFA b) Ambulatory = 10387 FCFA
10	Cobb *et al.* ^39^	2013	South Africa	Retrospective cohort	Cost analysis	Program	Bottom-up approach	WHO Nutritional care plans a) NCP-B for MAM b) NCP-C + NCP-B for SAM	Total= 113 MAM (88) SAM (25)	6 to 168	The cost per child (MAM) = $66.56 The cost per child (SAM) = $211.04
11	Colombatti *et al.* ^40^	2008	Guinea Bissau	Prospective cohort	Cost analysis	Health care provider	NR	Outpatient treatment + locally produced food	2642	51.6	The overall cost of the intervention was €13,448
12	Daga *et al.* ^41^	2010	India	Prospective cohort study	Cost analysis		Bottom-up	Treatment using drugs	111	1 to >60	The average cost per patient was $4
13	Fernandez *et al.* ^42^	1991	Peru	Observational	Cost analysis	Program	Bottom-up	Nutrition rehabilitation (education & child diet)	54	1 to 36	Cost per child = $21
14	Fronczak *et al.* ^43^	1993	Bangladesh	Cross sectional	Cost analysis	Program	Bottom-up	Nutritional rehabilitation	161	6 to 59	Average cost per child was $140
15	Garg *et al.* ^44^	2018	India	Randomized clinical trial	Cost analysis	Research & government	Price times quantity approach	Supplementary feeding: a) Centrally produced RUTF (RUTF-C) b) Locally produced RUTF (RUTF-L) c) Augmented, energy dense, home prepared food (A-HPF)	a) 124 b) 124 c) 123	6 to 59	Research costs per child: RUTF-C = $227 RUTF-L = $229 A-HPF = $238 Government costs: RUTF-C = $53 RUTF-L = $54 A-HPF = $61
16	Ghoneim *et al.* ^45^	2004	Egypt	Longitudinal, prospective	Cost analysis		Top-down approach	Nutrition rehabilitation (nutrition education and diet)	974	24 to 60	Cost per child per year was US$20.5
17	Glenn P Jenkins ^46^	2013	Uganda	Analytical modelling	Cost- effectiveness	Program	Modelling approach	Treatment with therapeutic feed	36907	-	Cost per child was $144.48 Cost per DALY gained was $36.27
18	Goudet *et al.* ^47^	2018	India	Cohort	Cost- effectiveness	Program & household	Activity-based costing	Aahar acute malnutrition programme vs standard care	12362	0 to 36	Cost per child was $27.11 Cost per death averted was $12360 Cost per DALY averted was $23
19	Greco *et al.* ^48^	2006	Uganda	Cohort	Cost analysis	Program	Bottom-up approach	Supplementary feeding (locally available Ingredients)	250–300	6 to 72	The low-cost porridge supplement (€2640/year/100 children)
20	Hoddinott *et al.* ^25^	2013	a) DRC b) Madagascar c) Ethiopia d) Uganda e) Tanzania f) Kenya g) Sudan h) Nigeria I) Yemen j) Nepal k) Bangladesh l) Pakistan m) India n) Vietnam o) Philippines p) Indonesia	Model study	Benefit cost ratios	Government	Modelling approach	Reducing stunting based on Bhutta *et al.* 2013 interventions			a) DRC = 3.8 b) Madagascar = 9.8 c) Ethiopia = 10.6 d) Uganda = 13 e) Tanzania = 14.6 f) Kenya = 15.2 g) Sudan = 23 h) Nigeria = 24.4 I) Yemen = 28.6 j) Nepal = 12.9 k) Bangladesh = 17.9 l) Pakistan = 28.9 m) India = 38.6 n) Vietnam = 35.3 o) Philippines = 43.8 p) Indonesia = 47.7
21	Hossain *et al.* ^49^	2009	Bangladesh	Cohort	Cost analysis	Hospital and program	Bottom-up approach	WHO recommendation(acute phase & nutritional rehab phase)	171	23.5 ± 15.3	Food= $6.1 Medicines= $8.5 Total(US$ 14.6 per child
22	Isanaka *et al.* ^50^	2016	Niger	Retrospective cohort	Cost analysis	Provider	Activity-based costing and Ingredients approach	Community-based treatment of SAM (CMAM, integrated)	16084	<60	Overall cost of the CMAM program = €148.86 per child a) Outpatient treatment cost = €75.50/child b) Inpatient treatment cost = €134.57/child c) Management and administration costs were €40.38/child
23	Isanaka *et al.* ^51^	2019	Mali	Cluster- randomized trial	Cost- effectiveness	Provider	Activity-based costing	Supplementary feeds: a) RUTF b) CSB++ c) Misola d) Locally milled flour vse) Treatment of SAM only	1264	6 to 35	Cost per child: a) $17.25 b) $8.10 c) $7.85 d) $8.50 e) 165Cost per DALY averted a) $347 b) $446 c) $490 d) $630 e) 142Cost per death averted a) $9821 b) $12435 c) $13146 d) $17486 e) 3974
24	Kielman *et al.* ^52^	1978	India	Longitudinal and cross-sectional	Cost- effectiveness	Program	Activity-based costing	a) Nutritional care (NUT) b) Medical care (MC) c) NUT + MC d) Control	2900	<36	Total service costs per child: a) NUT = $23 b) MC villages = $9 c) NUT *+* MC = $21 d) Control villages = $8 Cost per death averted: a) NUT = $76 b) MC = $135 c) NUT + MC = $21
25	King *et al.* ^53^	1978	Haiti		Cost analysis	Program	-	Centers for prevention and therapy for SAM			Total annual cost for the center = $4155 Cost per child is $10
26	Kittisakmontri *et al.* ^54^	2016	Thailand	Prospective cohort	Cost analysis	Hospital	Bottom-up approach	Hospitalization	53	1 to 59 Mean age (26.8 ± 1.8)	Total hospital expenditures for: a) Stunted children = €524.05 b) Wasted = €576.08 c) Stunted and wasted = €1175.58
27	Lagrone *et al.* ^55^	2010	Malawi	Prospective, observational	Cost analysis			Ready-to-use supplemental food	2417	6 to 59	Cost per child treated was $5.39
28	Lagrone *et al.* ^56^	2011	Malawi	Prospective, randomized, investigator blinded, controlled non- inferiority trial	Cost analysis	Provider	Bottom-up	a) Fortified blended flour (CSB++) b) Locally produced soy RUSF c) Imported soy/whey RUSF	a) 948 b) 964 c) 978	6 to 59	The cost of the three foods was as follows: US$0.03 for CSB++, US$0.04 for soy RUSF, and US$0.07 for soy/ whey RUSF per 100 kcal (418 kJ)
29	Loevinsohn *et al.* ^57^	1997	Philippines	Prospective study	Cost effectiveness	Government	Bottom-up approach	Vitamin A supplementation	a) Mild, moderate and severe malnutrition = 2,358,824 b) Moderate and severe = 398,450	6 to 59	Total costs: a) Mild, moderate and severe malnutrition = $1034510 b) Moderate and severe malnutrition = $888659 Costs per death averted: a) Mild, moderate and severe malnutrition = $144.1 b) Moderate and severe = $257.2
30	Marino *et al.* ^58^	2013	South Africa	Retrospective cohort	Cost analysis	Hospital	Bottom-up approach	Energy dense ready-to- use (RTU) infant feed vs fortified infant formula (PIF)	2652	<12 months	a) Energy dense RTU = €12.51 per day b) PIF + sunflower= €16.92 c) PIF + MCT oil = €19.61
31	Matilsky *et al.* ^59^	2009	Malawi	Randomized clinical effectiveness trial	Cost analysis	Provider	Bottom-up approach	Locally manufactured milk/peanut fortified spreads (FS) Soy/peanut FS Corn/soy blended flour (CSB)		6–60	The cost of the foods: Milk/peanut FS = US$0.16/1000 kJ Soy/peanut FS = US$ 0.08/1000 kJ CSB = US$0.04/1000 kJ
32	Medoua *et al.* ^60^	2016	Cameroon	Comparative efficacy trial	Cost analysis	Provider	Bottom-up approach	Ready-to-use supplemental food (RUSF) Corn–soya blend (CSB+)	81	25–59	Cost to treat a child with: CSB+ = €3.48 RUSF = € 3.52
33	Menon *et al.* ^61^	2016	India	Model study	Cost analysis	Program	Program experience approach	Community based management of SAM			Estimated cost per child was $200
34	Melville *et al.* ^62^	1995	Jamaica	Retrospective cohort	Cost analysis	Program	Bottom-up approach	Growth monitoring Community volunteer program	88	< 36	Total cost of the program in the two years was $2740. The total cost per child was $31.1
35	Moench- Pfanner ^30^	2016	Cambodia	Model study of economic losses due to malnutrition	Cost analysis	Government	Modelling approach	-	-	-	Economic losses due to: Wasting = $7.4 Underweight = $12.3 Stunting = $120.3
36	Ndekha *et al.* ^63^	2005	Malawi	Randomized controlled trial	Cost analysis	Provider	Bottom-up approach	RUTF	93	12 to 60	Cost per child $33
37	Nkonki *et al.* ^64^	2017	South Africa	Model study	Cost analysis	Provider perspective	Ingredients approach	Therapeutic feed and community-based treatment of MAM	-	-	Total costs: Therapeutic feeding = $12549660 Community based management of MAM = $28213620
38	Puett *et al.* ^65^	2013	Bangladesh	Cross-sectional	Cost- effectiveness	Societal	Activity-based costing	Community-based management of SAM delivered by community health workers (CMAM) vs inpatient treatment	1357	13 to 16	Cost per death averted: a) CMAM = $869 b) Inpatient = $45688 Cost per DALY averted: a) CMAM = $26 b) Inpatient = $1344 Cost per child treated: a) CMAM = $165 b) Inpatient = $1344 Cost per child recovered: a) CMAM = $180 b) Inpatient = $9149
39	Purwestri *et al.* ^66^	2012	Indonesia	Prospective cohort	Cost analysis	Institutional/ program	Bottom-up approach	Community-based daily program (semi urban area) vs weekly program (rural area)	204	Daily program (30.9 ± 12.9) Weekly program (31.6 ± 13.9)	Institutional costs (per child): a) Daily program = $234.3 ± 156.9 b) Weekly program = $257.1± 152.3 Total social costs (volunteer & caregivers time) per child: a) Daily = $141.9 ± 103.7) b) Weekly = $74.7 ± 54.8)
40	Qureshy *et al.* ^26^	2013	Indonesia	Modelling study	Cost-benefit analysis	Program	Modelling approach	Foetal and maternal growth monitoring, micronutrient supplements & immunizations *(Pyosandu*) and block grants ( *Generasi*)	306518		Total program cost is $114.8 million Cost per child = $ 18 Cost benefit ratio is 2.8
41	Rogers *et al.* ^67^	2018	Mali	Clinical cohort trial	Cost and cost effectiveness	Societal	Activity-based costing	a) CHW: screening in the community + referral to outpatient clinics b) CHW: outpatient clinics only	a) 617 b) 212	6 to 59	Cost per child: a) 244 b) 442
42	Rogers *et al.* ^68^	2019	Pakistan	Clinical cohort trial	Cost and cost effectiveness	Societal	Activity-based costing	a) LHW: screening in the community + referral to outpatient clinics b) LHW: outpatient clinics only	a) 425 b) 393	6 to 59	Cost per child: a) 291 b) 301
43	Rogers *et al.* ^69^	2019	Pakistan	Randomized controlled trial	Cost and cost effectiveness	Institutional	NR	a) SAM treatment only b) SAM treatment + Aquatabs c) SAM treatment + flocculent disinfection d) SAM treatment + ceramic filters	901	6 to 59	Cost per child treated: a) 256 b) 239 c) 290 d) 369 Cost per child recovered: a) 482 b) 318 c) 416 d) 522 ICER (Aquatabs vs SAM treatment only) = $24
44	Sandige *et al.* ^70^	2004	Malawi	Randomized controlled trial	Cost analysis	Provider	Bottom-up approach	a) RUTF (local) b) RUTF (imported)	260	12 to 60	Cost per child: a) $22 b) $55
45	Sayyad-Neerkorn *et al.* ^71^	2015	Niger	Prospective cohort	Cost analysis	Provider	Bottom-up approach	a) SC+ b) LNS	a) 845 b) 1122	a) 17.4 b) 15.2	Cost per child: a) 154.8 b) 121.05
46	Shekar *et al.* ^28^	2016	DRC, Mali, Nigeria and Togo	Modelling study	Cost effectiveness	Government	Program experience approach	Cost of scaling up 10 Lancet interventions (Bhutta 2013)			Cost per DALY averted: DRC = $143 Mali = $ 178 Nigeria = $ 141 Togo = $127 Cost per life year saved: DRC = $226 Mali = $ 344 Nigeria = $ 292 Togo = $238
47	Tekeste *et al.* ^72^	2012	Ethiopia	Retrospective cohort	Cost- effectiveness	Societal perspective	Bottom-up approach	Community-based therapeutic care (CTC) vs therapeutic feeding (TFC)	306	CTC (41.42 ± 20.58) TFC (59.4 ± 47.8)	The total cost per child treated: a) CTC = $134.88 b) TFC = $284.56 Total institutional costs per child: a) TFC = $262.62 b) CTC = $128.58 Caretakers cost per child: a) CTC = $6.29 b) TFC = $ 21.93
48	Waters *et al.* ^73^	2006	Peru	Prospective	Cost effectiveness	a) Provider b) Household	Activity-based costing	Nutrition education programme	187	0 to 18	Cost per child: a) $15.37 b) $0.46 Cost per case averted = $ 138.50 Cost per death averted = $1952
49	Whittaker *et al.* ^74^	1985	South Africa	Retrospective cohort	Cost analysis	Program	Modelling approach	Philani Nutrition day center for rehabilitation of undernourished children (SAM and MAM)	42	0 to 84	Total costs = R29759 Overall cost per child/attendance= R2.42 Cost per child a) SAM = R194 b) MAM = R73
50	Wilford *et al.* ^75^	2011	Malawi	Decision analytical modelling	Cost- effectiveness	Program & government	Modelling approach	CMAM integrated into existing health services (CMAM) vs non-CMAM	2780	<60	Cost per DALY averted (CMAM) = US$42 Cost per life saved (CMAM) = US$1365 Total cost for providing: a) CMAM cost was $470,703 b) Non-CMAM cost was $23,394

SAM, severe acute malnutrition; NR, not reported; RUSF, ready-to-use supplementary food; CSB, Corn-Soy Blend; LMF, locally milled flours; CTC, community-based therapeutic care; DALY, disability-adjusted life year; PEM, protein energy malnutrition; NCP, nutritional care plan; MAM, moderate acute malnutrition; RUTF, ready-to-use therapeutic food; A-HPF, augmented, energy dense, home prepared food; DRC, Democratic Republic of the Congo; CMAM, community-based management of acute malnutrition; NUT, nutritional care; MC, medical care; PIF, powdered infant formula; SC, Super Cereal; LNS, lipid-based nutritional supplement; FS, fortified spread; MCT, medium-chain triglyceride; CHW, Community Health Worker; LHW, Lady Health Worker; TFC, therapeutic center; ICER, incremental cost-effectiveness ratio.

### Year of publication

The included articles were published between 1972 and 2019, with majority (66%) published from 2009. Of those published from 2009, 17 assessed the cost of supplementary feeds administered to children with MAM, while twelve studies assessed costs of implementation of CMAM programs in different regions, four of which compared CMAM to facility-based care of children with SAM. Studies published between 1972 and 1997 mainly focused on nutritional rehabilitation programs involving administration of supplementary feeds or special diets to children, parental counselling and monitoring. Two of these studies assessed the cost of inpatient treatment for children with malnutrition.

### Studies by region and continent

**Figure 2.**
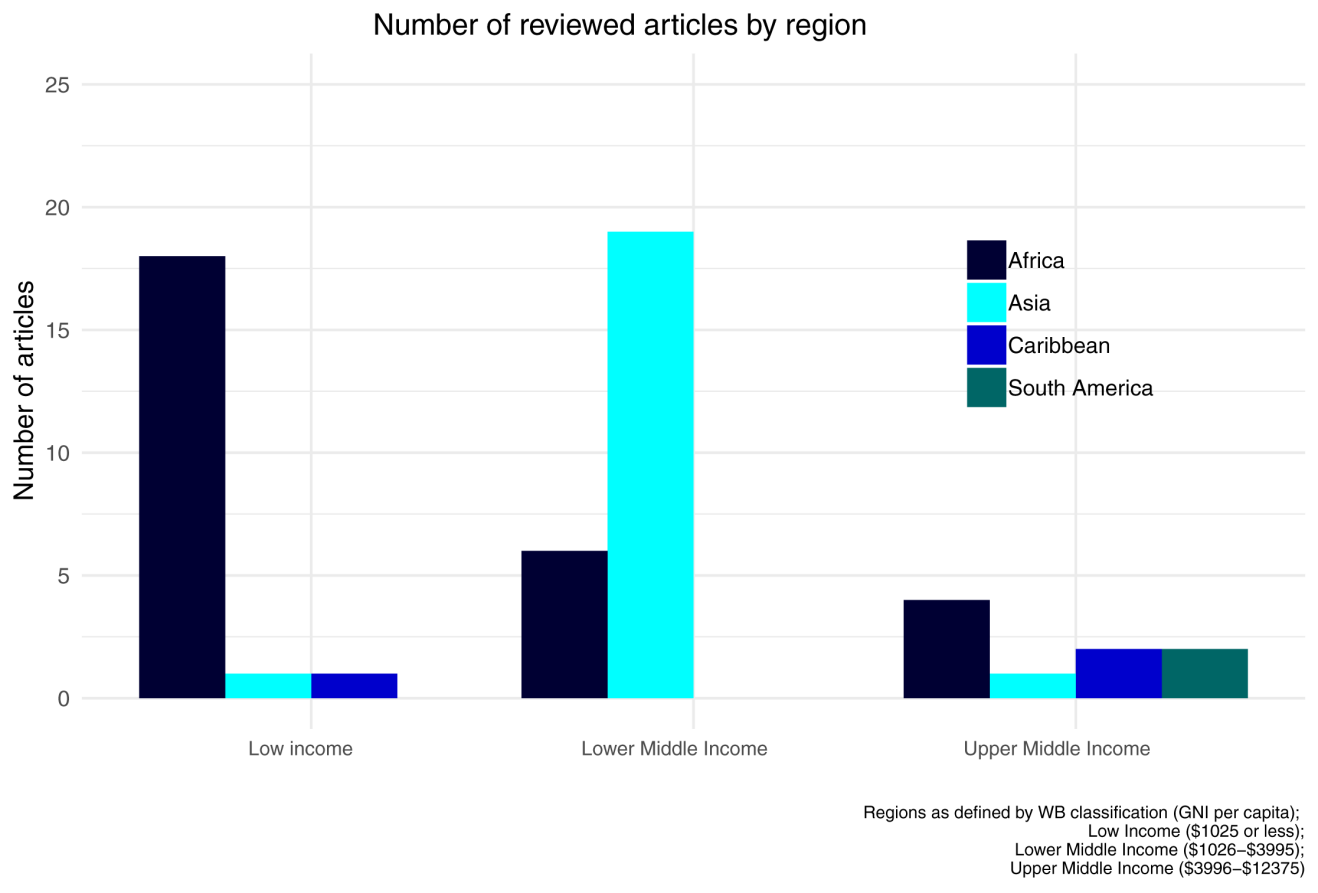
Number of articles by World Bank classification regions WB, World Bank; GNI, gross national income.

Overall, most studies were carried out in Africa (56%) and Asia (34%), while others were done in the Caribbean and South America ( Figure 2). With reference to the World Bank classification of countries ^24^, more than 75% of these studies were conducted in either low-income or lower middle economies (with Gross National Income per capita of less than $3996).

### Perspective of the analysis

**Figure 3.**
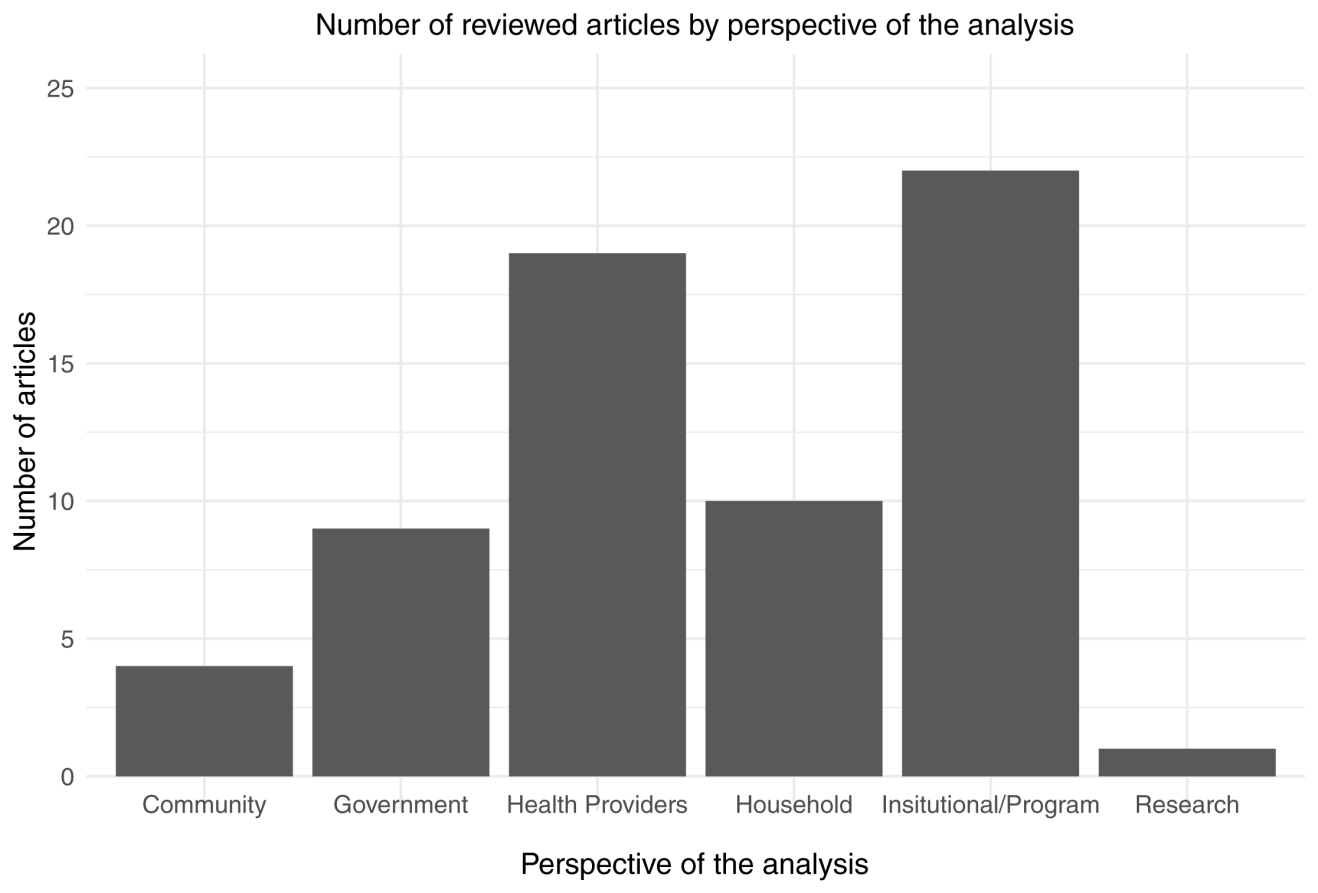
Number of articles by perspective of the analysis.

Perspective in economic evaluation describes the viewpoint adopted when deciding the scope of costs and benefits to be included ^21^. Studies in this review mostly adopted an institutional/program perspective (44%) or health provider perspective (38%) ( Figure 3). Nine studies reported costs from the government’s perspective, three of which modelled the costs of scaling up nutrition interventions to reduce stunting. Only ten studies included in this review assessed costs incurred during treatment of child undernutrition from more than one perspective ( Table 2).

### Type of economic evaluation and analytical approach

Studies included were cost analyses (n=33), cost-effectiveness studies (n=15) and cost benefit analyses (n=2). The cost analysis approach only measures costs without considering outcomes. The cost-effectiveness technique measures relative cost against effectiveness of the intervention, while cost-benefit analysis compares cost of intervention against benefits gained from the intervention. Eight of the cost-effectiveness analysis studies assessed the standard CMAM program compared to alternative treatment. The two cost-benefit analysis studies reported cost benefit ratios of interventions aimed at reducing stunting ^25, 26^.

**Figure 4.**
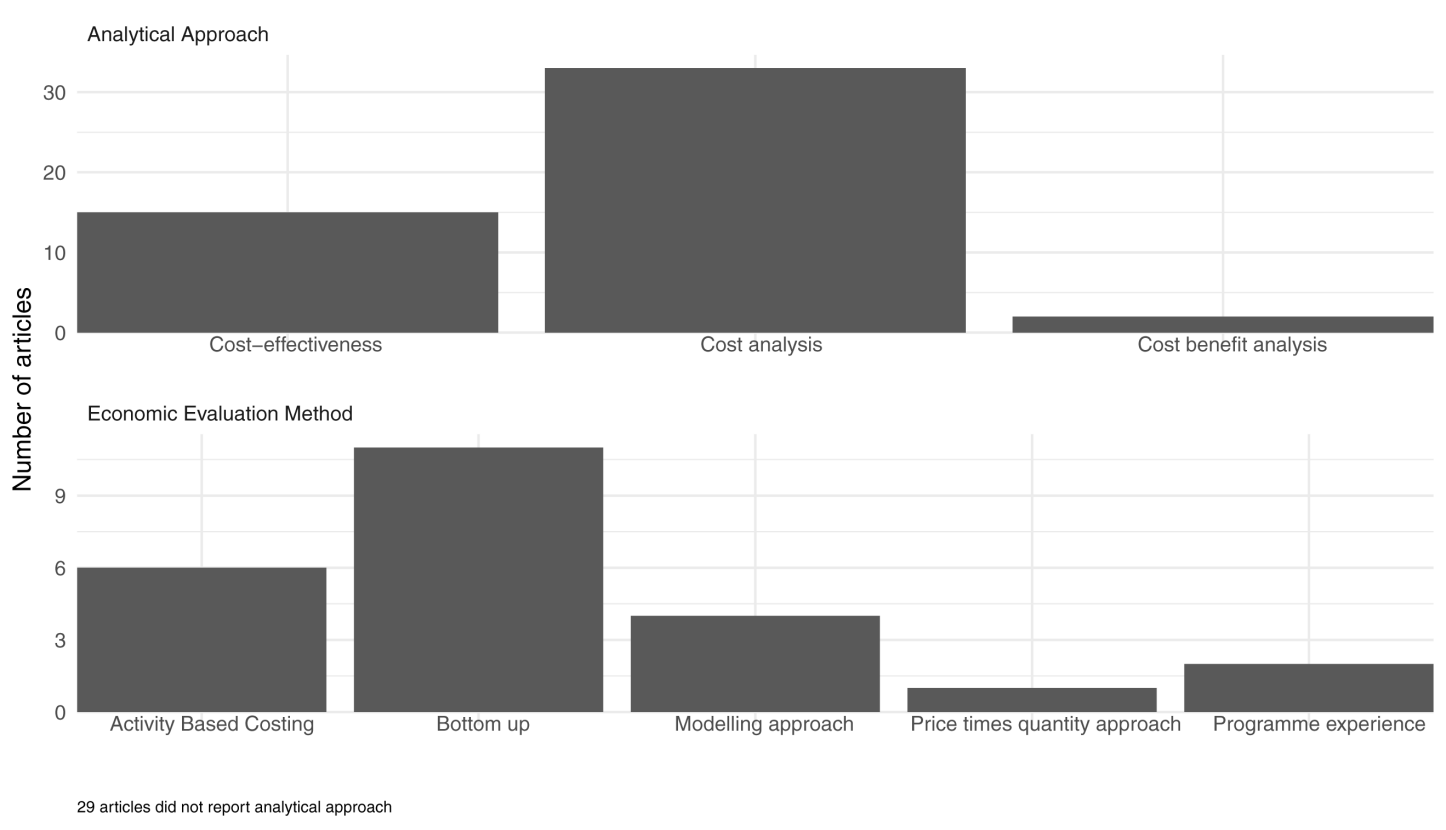
Number of articles by type of economic evaluation and analytical approach.

The majority (22%) of these studies adopted the bottom-up approach to costing, while program experience and price times quantity approaches (6%) were the least used ( Figure 4). The bottom-up approach estimates total costs through the multiplication of unit costs by the quantities used ^27^. The programme experience approach utilizes cost data for each intervention from actual programs in operation while considering the delivery channels ^28^. Activity-based costing involves assignment of costs to departments or activities then to various services ^21^.

### Economic evaluation by perspective

***Government perspective.*** We defined this as costs incurred by the government for treatment of child undernutrition. We identified nine studies reporting these costs. Five of these studies modelled the economic consequences of undernutrition and the cost of scaling up stunting interventions in African and Asian countries. Among these, two studies explored the economic losses in Cambodia associated with 14 nutrition indicators of malnutrition including stunting, underweight and wasting ^29, 30^. The studies used a consequence model to estimate the value of economic losses due to increased child mortality, depressed future productivity, and excess healthcare expenditures attributable to malnutrition. On average, losses due to malnutrition accounted for more than 260 million USD annually; equivalent to approximately 1.5% of the Cambodian GDP. Notably, average annual losses due to stunting was higher (US$124 million) compared to underweight (US$17 million) and wasting (US$13 million). This was due to the high prevalence of stunted children in the country.

A study published in 2013 assessed the cost benefit analysis of interventions aimed at reducing stunting for 17 high burden countries ^25^. The benefit cost ratio for all the countries was greater than one and ranged between 3.5 (Democratic Republic of the Congo, DRC) to 48 (Indonesia), meaning that an equivalent of $US3.5 and $US48 in economic returns could be generated in DRC and Indonesia, respectively, for every dollar invested in programmes aimed at reducing stunting.

Cost-effectiveness analyses of nutrition-specific interventions was conducted using data from four African countries ^28^. The cost per DALY averted ranged between (US$127–US$178), which was below the established willingness to pay threshold in these countries, suggesting that scaling up these interventions was cost effective.

One study explored costs borne by the government during the implementation and integration of a CMAM program into existing health services ^75^. Findings from this study showed that the government covered only 10% of the total costs. These included administrative costs, inpatient costs for children who were referred to inpatient treatment and labor costs by the clinic staff and supervisors. The main driver of these costs were labor costs (US$12 per child).

***Community volunteers perspective.*** We defined this as the direct and indirect costs incurred by community volunteers during the implementation of CMAM. The review identified five studies assessing these costs ^31, 65– 68^. Two studies conducted in Mali and Pakistan compared the cost effectiveness of treatment of uncomplicated SAM by community health workers (CHWs) to outpatient facility based programs ^67, 68^. The study in Mali reported that delivery of treatment by CHWs ($259 per child recovered) was more cost-effective compared to the outpatient facility care ($501 per child). The study in Pakistan, however, reported considerable uncertainity as to which method was more cost-effective as results of the sensitivity analyses showed small differences in costs and recovery rates between the two arms ( Table 3). In addition, a paper done in Bangladesh assessing the cost-effectiveness of CMAM delivered by CHWs found out that this was more cost-effective (US$26 per DALY averted) than inpatient treatment (US$1344 per DALY averted). Each CHWs was paid a monthly stipend of US$11.80 during this study ^65^

**Table 3.** Costs and cost-effectiveness of community-based management of severe acute malnutrition (CMAM integrated programs).

	Author; year	Country	Sample size (n)	Intervention	Outcome	Cost per child (USD)	Cost per DALY averted/ gained (USD)	Cost per life year saved (USD)	Cost per death averted (USD)
1.	Abdul-Latif 2014 ^31^	Ghana	40	CMAM	NR	805	NR	NR	NR
2.	Bachmann 2009 ^35^	Zambia	2523	a) CMAM b) Hypothetical no treatment	Mortality: a) 9.2% b) 20.8%	203	53 (DALY gained)	1760	NR
3.	Goudet *et al.* 2018 ^47^	India	12362	a) Aahar acute malnutrition program b) Standard of care	Cured	27	23		12360
4.	Isanaka *et al.* 2016 ^50^	Niger	16084	CMAM	NR	196	NR	NR	NR
5.	Isanaka *et al.* 2019 ^51^	Mali	1264	Treatment of MAM: a) RUTF b) CSB++ c) Misola d) Locally milled flour Treatment of SAM only	Reduced risk of death: a) 15.4% b) 12.7% c) 11.9% d) 10.3% SAM: NR	a) 17.25 b) 8.10 c) 7.85 d) 8.50 SAM: 165	a) 347 b) 446 c) 490 d) 630 SAM: 142	NR	a) 9821 b) 12435 c) 13146 d) 17486 SAM: 3974
6.	Puett *et al.* 2013 ^65^	Bangladesh	1357	a) CMAM b) Inpatient treatment (“standard of care”)	Recovery rates: a) 91.9% b) 1.4%	a) 165 b) 1344	a) 26 b) 1344		a) 869 b) 45688
7.	Purwestry *et al.* 2012 ^66^	Indonesia	a) 103 b) 101	a) CMAM (daily supervision) b) CMAM (weekly supervision)	Weight gain: a) 3.7g/kg/day b) 2.2g/kg/day	a) 376 b) 331	NR	NR	NR
8.	Rogers *et al.* 2018 ^67^	Mali	a) 617 b) 212	a) CHW: screening/ treatment in community + referral to outpatient clinics b) CHW: outpatient clinics only	Recovery rates: a) 94.17% b) 88.21%	Cost per child treated a) 244 b) 442 Cost per child recovered: a) 259 b) 501	NR	NR	NR
9.	Rogers *et al.* 2019 ^68^	Pakistan	a) 425 b) 393	a) LHW: screening/ treatment in community + referral to outpatient clinics b) LHW: outpatient clinics only	Recovery rates: a) 76% b) 82.3%	Cost per child treated: a) 291 b) 301 Cost per child recovered: a) 382 b) 383 ICER (control): 146	NR	NR	NR
10	Rogers *et al.* 2019 ^69^	Pakistan	901	a) SAM treatment only b) SAM treatment + Aquatabs c) SAM treatment + flocculent disinfection d) SAM treatment + ceramic filters	Recovery rates a) 53.1% b) 75.2% c) 69.7% d) 70.7%	Cost per child treated: a) 256 b) 239 c) 290 d) 369 Cost per child recovered: a) 482 b) 318 c) 416 d) 522 ICER (Aquatabs) = $24			
11	Tekeste *et al.* 2012 ^72^	Ethiopia	306	a) CMAM b) Facility-based therapeutic care	Cure rates a) 94.3 % b) 95.36%	a) 135 b) 285	NR	NR	NR
12	Wilford *et al.* 2011 ^75^	Malawi	2780	a) CMAM integrated into existing health services b) Existing health services (inpatient care)	Mortality a) 11.9% b)17.1%	a) 165 b) 16.7	a) 42	a) 1365	NR

DALY, disability-adjusted life year; USD, United States Dollars; NR: not reported; CMAM, community-based management of malnutrition; LHW, Lady Health Worker; CHW, Community Health Worker; RUTF, ready-to-use therapeutic feeding; SAM, severe acute malnutrition; CSB, corn soy blend; ICER, incremental cost-effectiveness ratio.

The other two studies conducted in Ghana ^31^ and Indonesia ^66^ reported indirect and transport costs incurred by community volunteers while implementing the CMAM program. The average costs were US$61 and $0.2 per child for indirect costs and transport costs, respectively.

***Household perspective.*** We defined this as the direct and indirect costs incurred by families of children with undernutrition. Ten studies conducted between 1997 and 2019 reported costs from the household’s perspective. Nine studies considered interventions for children under the age of five years with SAM. The average cost per child to households ranged widely from US$0.5 in Peru ^73^ to US$82 in Bangladesh ^65^. The least costly study in Peru (2006) involved a nutritional education programme in which the households only incurred transportation and consultation costs; all other costs were incurred by the health facilities delivering the program. The Bangladesh study (2016) compared costs incurred during CMAM and inpatient treatment, with the latter being more costly to the households (US$82) per child treated.

Overall, the least costly treatments to households were those involving outpatient management, day care or CMAM programs, costing US$0.5–US$69 per child compared to traditional inpatient management (US$3.1–US$538). Among the direct medical costs, supplementary feeds was the highest cost driver ($14 per child) to the households, as reported by a study conducted in Ghana during the implementation of a CMAM program ^31^. Productivity loss was also higher in inpatient care than outpatient care due to the longer periods spent in health care facilities with their children during treatment ( Table 4). Overall, direct non-medical costs such as food (US$32) and indirect costs (US$21) were the main cost drivers to households.

**Table 4.** Cost per child per treatment in USD incurred by households.

	Outpatient (CMAM, day care, domiciliary care)			Inpatient management		
Cost categories	Mean (SD)	Median [IQR]	N *	Mean (SD)	Median [IQR]	N *
Direct medical costs						
Medication costs	-	-		7.6	7.6	1
Supplementary feeding	14.4	14.4	1	-	-	-
Administrative costs	0.4	0.4	1	-	-	-
Direct non-medical costs						
Transport costs	1.9 (1.6)	2.0 [0.7,2.4]	4	2.9 (3.8)	0.9 [0.7-4.1]	3
Food (non-medical)	6.6 (7.5)	4.0 [3,6]	4	32.1	32.1	1
Indirect costs (loss of income)	18.9 (24.5)	10.2 [3,22]	6	16.6 (12.4)	21.0 [11-23]	3

USD, United States Dollars; CMAM, community management of acute malnutrition; SD, standard deviation; IQR, interquartile range; N*, number of articles included.

***Health providers’ perspective.*** We defined this as the direct medical and direct non-medical costs incurred by institutions offering health services. Of the included studies, 19 reported costs from the health provider’s perspective. These studies assessed costs incurred due to provision of supplementary feeds for children with MAM, cost of outpatient treatment (CMAM, daycare management and domiciliary management) and costs of inpatient care. Costs borne by the providers included both direct medical and direct non-medical costs ( Table 5). The average cost per child per treatment ranged widely between the studies (US$4-US$811.31). The main driver of costs for the health providers were personnel costs (personnel wages and salaries).

**Table 5.** Cost per child per treatment in USD incurred by health providers.

Cost categories	Mean (SD)	Percentage of total mean costs	Median [IQR]	N *
Direct medical costs				
Personnel costs	117 (226)	50	35 [8-99]	6
Medication costs	42 (65)	18	20 [9-41]	6
Capital costs	18 (13)	7	19 [8-28]	3
Administrative costs	18 (25)	7	2 [1-34]	3
Supplementary feeding	29 (36)	12	16 [8-34]	14
Direct non-medical costs				
Transport costs	9 (16)	3	0.6 [0.3-14]	3

USD, United States Dollars; SD, standard deviation; IQR, interquartile range; N*, number of articles included.

***Program perspective.*** We defined this as the direct medical and direct non-medical costs incurred by non-health care organisations and institutions implementing programs aimed at managing child undernutrition. In total, 22 articles reported these costs. These programs included community-based management of malnutrition and nutrition rehabilitation centers set up for children with malnutrition. Costs incurred by these organizations included direct medical and direct non-medical costs ( Table 6). The costs incurred ranged from US$0.15 to US$449.56. The main drivers were personnel costs (personnel wages and salaries) and administrative costs (training costs, monitoring and mobilization costs).

**Table 6.** Costs per child per treatment in USD incurred by institutions/programs.

Cost categories	Mean (SD)	Percentage of total mean costs	Median [IQR]	N *
Direct medical costs				
Personnel costs	120 (139)	35	107 [23–160]	12
Medication costs	33 (65)	9	4 [2–20]	5
Capital costs	28 (40)	8	15 [4–18]	9
Administrative costs	79 (138)	23	20 [12–35]	5
Supplementary feeding	45 (50)	13	42 [5–64]	15
**Direct non-medical costs**				
Transport costs	31 (44)	9	24 [2–29]	4
Food (non-medical)	6 (4)	1	5 [2–10]	2

USD, United States Dollars; SD, standard deviation; IQR, interquartile range; N*, number of articles included.

### CMAM

The costs and cost-effectiveness of CMAM integrated programs for treatment of children under five with SAM were assessed in 12 studies published after 2009; seven of these were implemented in African countries and five in Asian countries. These costs included; personnel, supplementary feeding, transport and opportunity costs to households and community volunteers. The costs ranged from $135 in Ethiopia to $850 per child in Ghana. The main drivers of costs incurred were personnel costs, which were as high as $200 per child in Indonesia, and supplementary feeds, which ranged from $13 to $87 per child, the least costly feeds being made from locally available materials.

Additionally, four studies assessed the cost-effectiveness of the CMAM program ^35, 65, 72, 75^. Cost per disability adjusted life year (DALY) for the CMAM program ranged between US$26 and US$53, which was much lower compared to facility-based management (US$1344 per DALY averted) ( Table 3). Further, a study carried out in Malawi reveals that integration of a community-based program into existing health services is cost-effective ^75^. The study used a decision tree model to compare costs and effects of existing health services with CMAM and existing health services without CMAM. In this study, there were 342 less deaths in the CMAM implemented scenario compared to the non-implemented scenario. The resulting cost per DALY averted for adding CMAM in to existing health services was US$42, which was highly cost-effective.

Overall, cost per child for the CMAM programs implemented by community volunteers was $216 while CMAM implemented in traditional facility-based programs was $300 per child ( Table 3).

### Productivity loss and coping strategies

In addition to direct health care costs such as drug costs and transport costs incurred by households due to malnutrition, families spend a lot of time away from their normal duties to seek treatment. Findings from one retrospective study done in rural Ghana to assess the costs of CMAM revealed that high costs were incurred by families to ensure normal running of household’s activities while seeking treatment ^31^. More than a third of the total household costs constituted the cost of employing people to take care of what the caregivers would have been doing if they were not seeking care. This was equivalent to US$16 per child treated in the program.

In addition, the huge financial burden to households leads to different coping mechanisms being adopted to mitigate necessary payment for healthcare for their children. A study done in Bangladesh reported that some of the households received food as gifts from their relatives and neighbours in order to meet the prescribed dietary requirements for their children after treatment ^34^.

### Quality assessment of the studies

Among the 17 items in the GHCC guidelines ( Table 7), only nine items were either partially or fully met by more than 60% of the included studies. For instance, of the 50 studies, less than half stated the costing methods used and perspective of the analysis, which are important components in economic evaluations according to the guidelines. Further, only 18 studies conducted sensitivity analysis to characterize any uncertainity in the reported cost estimates.

**Table 7.** Quality assessment of studies as highlighted in Global Health Cost Consortium (GHCC).

		Number of articles (%)		
	Principle	1=Satisfied	0=Not satisfied	Not applicable*
	Study design and scope			
1	Purpose, population & intervention	50 (100)	0 (0)	0 (0)
2	Perspective	22 (44)	28 (56)	0 (0)
3	Type of cost	29 (58)	21 (42)	0 (0)
4	Unit costs	46 (92)	4 (8)	0 (0)
5	Time (Data year/Time horizon)	50 (100)	0 (0)	0 (0)
	Service use and resource use measurement			
6	Scope of inputs	41 (82)	9 (18)	0 (0)
7	Costing method (costing approach)	21 (42)	29 (58)	0 (0)
8	Sampling strategy	50 (100)	0 (0)	0 (0)
9	Selection of data source	35 (70)	15 (30)	0 (0)
10	Timing of data selection (prospective/retrospective)	41 (82)	9 (18)	0 (0)
	Valuation and pricing			
11	Sources of price data	34 (68)	16 (32)	0 (0)
12	Amortization of capital costs	11 (11)	21 (30)	17(59)
13	Discounting, inflation (where relevant)	10 (20)	23 (46)	17 (34)
14	Use of shadow prices	9 (18)	6(12)	35 (70)
	Analyzing and presenting results			
15	Heterogeneity	22 (44)	28 (56)	0 (0)
16	Sensitivity analysis	18 (36)	32 (64)	0 (0)

## Discussion

This review gives a breakdown of direct and indirect costs borne by households, health providers, the community, institutions/programs and the government. The studies varied in the interventions studied and costing methods used, with studies reporting treatment costs between US$0.44 and US$1344 per child. The majority of the included studies were done in Africa and Asia. This could be explained by the high burden of child undernutrition in these regions ^7^, leading to numerous efforts to manage its cost and health implications. In line with the WHO recommendations on management of child undernutrition ^76^, included studies assessed interventions such as supplementary feeding for children with moderate acute malnutrition, nutritional rehabilitation and community management of severe acute malnutrition. Most included studies adopted the institutional/program (44%) and health provider (38%) perspectives, while only four adopted the community volunteers’ perspective.

Integration of outpatient and inpatient care for children with undernutrition was recommended after endorsement of CMAM in 2007. However, most of the studies reviewed compared cost outcomes of outpatient and inpatient care separately. This review identified only one study conducted in Malawi ^75^ assesing the costs of integrating CMAM into existing health services, concluding that it is cost-effective (US$42 per DALY averted). For generalizability and strengthening of this evidence to inform policy, there is need to conduct similar studies from a range of settings to assess cost-effectiveness of integrating CMAM into primary healthcare. 

According to this review, substantial costs for health providers and programs were due to personnel, medication and therapeutic feeds. The costs of therapeutic feeds were high mainly because they were imported. This suggests that production of feeds using local ingredients could potentially reduce costs. Studies reporting from these perspectives mainly assessed the costs of implementing the CMAM program, whose key components are administration of supplementary feeds and involvement of CHWs for community mobilization ^11^ to ensure high coverage and timely detection of children with malnutrition.

Despite a major role played by CHWs during the implementation of CMAM, only two studies included in this review assessed the costs they incurred. This included transport costs ($0.2 per child) and indirect costs, which were as high as US$60 per child ^31, 66^. In these studies, compensation to the volunteers was done by the funding organisations only in form of food and household goods. These findings imply that to ensure effective and efficient implementation of the CMAM program in future, there is a need to consider more structured and better compensation methods for CHWs. This is in support of findings from a study conducted in Mali assessing the cost-effectiveness of treatment of uncomplicated SAM using CHWs and outpatient facilities. In this study, treatment using CHWs was cost-effective ^67^.

In addition to the out of pockets costs incurred by families with children affected by malnutrition, this review reveals that indirect costs were the main driver of costs, especially for those admitted to hospital. This could be explained by the longer duration of time spent away from normal duties to take care of children, resulting in lost income. This highlights the need for adoption of the CMAM program in more countries, which would contribute to early identification and treatment of malnutrition cases to avoid worsening of illness and prolonged inpatient hospital stays. In addition, medication costs incurred by families were also high, especially for children with SAM. This was mainly due to co-infections associated with acute malnutrition ^77^. Supplementary feeds and transport costs were also significant costs incurred by families due to undernutrition. Although feeds were mostly provided by organizations, the cost of preparing them fell on the caregivers. For instance, a third of total household costs in a study conducted in Ghana constituted the cost of preparing these feeds ^31^.

These costs highlight the huge financial implications to households attributable to undernutrition. For poor households, especially in low-income settings, this could be catastrophic as they are less equipped to endure the adverse impact on their income ^78^. This may result to borrowing from friends and family members, selling of assets and reliance on well-wishers as coping strategies towards these costs. Interviews conducted in households in rural Ghana indicated that families of children with malnutrition resulted in; cheaper treatment options for their sick children other than professional healthcare, reliance on other family members to pay medical costs and reliance on non-profit organizations for both food and medication. This was mainly due to lack of reliable sources of income for the parents ^79^. This highlights the need to identify affordable interventions for prevention and treatment of malnutrition in children, especially in these settings.

Additional findings from this review support previous findings that governments incur huge costs due to malnutrition ^80^. However, a study included in this review shows that investing in a set of nutritional interventions to reduce stunting is beneficial ^25^. The study showed that investing at least one dollar to reduce stunting could generate an average of US$18 worth of benefits in LMICs. This is consistent with findings from a previous review providing evidence of a reduction of 15% mortality due to stunting in children under five years if interventions were accessible at 90% coverage.

### Limitations

This review had certain limitations. First, heterogeneity in the costing methods, interventions assessed and reporting of costs precluded a comprehensive comparison of costs and therefore, meta-analysis was inappropriate. Secondly, A limitation inherent in the available data was that there was a wide range of cost outcomes and unit measurements for some of the outcomes, cost categories for similar cost centres varied a lot among the studies. Thus, meta-analysis was inappropriate.

Thirdly, from our quality assessment of the included studies, less than half of the items on the GHCC guidelines were either partially or fully met by the included studies. For instance, most articles did not mention the perspective, costing approach used and did not conduct sensitivity analysis to characterise uncertainties in the reported costs outcomes Lastly, full texts that were neither in English nor French were not included in the review. Therefore, some relevant evidence might have been missed.

## Conclusions

Integration of outpatient and inpatient care for children with undernutrition through the CMAM program has been recommended as it is more effective and cost-effective compared to traditional programs characterised by prolonged inpatient duration. However, this review reveals that many countries have not adopted the integrated CMAM program, hence studies still report cost outcomes of inpatient and outpatient care separately. This highlights the need for more countries to adopt the CMAM program to reduce cost implications. Further, cost studies need to shift towards evaluating integrated programs to provide insight into different and more cost-effective ways of delivering the CMAM program through primary healthcare.

Additionally, current cost estimates on integrated programs include substantial support from international organisations which may represent higher costs. Therefore, there is need for more studies to generate cost estimates of integrated programs from government delivered programs to represent the actual situation.

This review also reveals the paucity of data on the economic burden of undernutrition to households and communities. More studies are needed to assess this burden in order to assist in planning, identifying cost-effective solutions and addressing issues of cost that may limit delivery, uptake and effectiveness of interventions.

We also recommend that for easy and comprehensive secondary analysis all items as listed in the GHCC guidelines including explicitly stating the perspective of the analysis, costing methods used, conducting sensitivity analysis should be adhered to by authors. Further, for comprehensive comparison of the cost and cost-effectiveness of interventions or treatments used in studies, this review recommends a standardization of methods used and cost categories reported in economic evaluations as per the GHCC guidelines.

## Data availability

### Underlying data

Figshare: Cost and cost effectiveness analysis of treatment for child undernutrition in low and middle income countries: A systematic review-Dataset https://doi.org/10.6084/m9.figshare.11985873.v2 ^81^

This project contains the following underlying data:

- Dataset in CSV format
- Data code book in PDF format

### Reporting guidelines

Figshare: Cost and Cost effectiveness Analysis of Treatment for Child Undernutrition in Low and Middle Income Countries: A Systematic Review-PRISMA Checklist https://doi.org/10.6084/m9.figshare.11961153.v2 ^82^

Data are available under the terms of the Creative Commons Attribution 4.0 International license (CC-BY 4.0).
