# Supplementary material for: Cost and cost-effectiveness analysis of treatment for child undernutrition in low- and middle-income countries: A systematic review
Source: Wellcome Open Res. 2020 Oct 5;5:62. Originally published 2020 Apr 3. [Version 2] doi: 10.12688/wellcomeopenres.15781.2 (PMC7569484; doi:10.12688/wellcomeopenres.15781.2)
Supplement: Peer review history

# Peer review: Reviewer response for version 2

**DOI:** 10.21956/wellcomeopenres.17897.r40709

**Published:** 2020-10-14

**Authors:** Chloe Puett

**Affiliations:** 1 Stony Brook University, New York City, USA

The authors have adequately addressed all of my comments and the revised version of the manuscript is acceptable to me.

Are the rationale for, and objectives of, the Systematic Review clearly stated?

Partly

Is the statistical analysis and its interpretation appropriate?

Not applicable

Are sufficient details of the methods and analysis provided to allow replication by others?

Yes

Are the conclusions drawn adequately supported by the results presented in the review?

Yes

Reviewer Expertise:

Economic analysis of nutrition interventions

I confirm that I have read this submission and believe that I have an appropriate level of expertise to confirm that it is of an acceptable scientific standard.

# Peer review: Reviewer response for version 1

**DOI:** 10.21956/wellcomeopenres.17307.r38354

**Published:** 2020-05-07

**Authors:** Chloe Puett

**Affiliations:** 1 Stony Brook University, New York City, USA

This manuscript presents a systematic review of the under-researched area of economic analysis of nutrition interventions in LMICs, addressing an important gap in the scientific literature. The authors give special attention to costs incurred by households during treatment, which is an often-ignored aspect of economic analysis for nutrition with real implications for intervention coverage, adherence and, ultimately, effectiveness. The review appears to have been well-conducted and the analytical approach is described in detail. However there are a few points of clarification needed that would help position this article's contributions more specifically relative to the evidence that it presents.

 The objective of the analysis in the introduction is stated as "[determining] the current state of knowledge on costs of child undernutrition treatment to households, health providers, organizations and governments in low and middle-income countries (LMICs)." In the methods section, the inclusion/exclusion criteria section simply states that articles were included that "involved undernutrition or interventions related to undernutrition" and that articles were included that "reported global cost estimates of child undernutrition *treatment*", and goes on later to specify that this focused on anthropometry outcomes and excluded micronutrient deficiencies. The exact inclusion criteria (and preferably the justification for this focus) should be clarified in the introduction. As part of this, the authors should specify what is meant by "treatment" and clearly describe what interventions were/were not under consideration, again with justification if possible. It would be interesting and informative, for example, to know why the authors did not include costs of prevention in the search.

 Given that the introduction refers to stunting outcomes, one wonders why the keywords for stunting (and underweight) and related terms (height-for-age (HAZ), weight-for-age (WAZ), stuntedness, etc) were not included in the search terms listed in Table 1. The included terms would seem to position the paper to be more of a review on economic analysis of acute malnutrition than undernutrition more generally, including chronic undernutrition. 

 Perhaps due to this oversight in search terms (if my understanding is correct), at least one potentially relevant study does not appear to be included in the review:

 Alderman H *et al*. (2017). Big numbers about small children: Estimating the economic benefits of addressing undernutrition. *The World Bank Research Observer*, 32(1), 107-125 ^1^.

 p. 18: For the approaches to costing, it should be clarified which of these approaches use institutional accounting data (instead of using unit costs and quantities alone via an 'ingredients' approach). 

 p. 18: In addition to the point above, I would recommend that in the sub-section "economic evaluation by perspective" that the authors first define and describe each perspective analyzed and presented. For example it is currently unclear what is the difference between the health providers and institution/program perspective. And when the authors describe the "community volunteer perspective" are they referring to studies which include direct and indirect costs incurred by community volunteers during implementation (which would seem to be more an aspect of a general societal perspective), or is this a broader analytical perspective? 

 p. 18: Puett *et al* 2013 also considers costs for CMAM delivered by community volunteers, and includes the indirect costs of their time allocation in the analysis

 pp 20-21: Would it be possible for the authors to break down costs of CMAM by programs implementing traditional facility-based CMAM versus programs delivered by community volunteers (i.e. a community case management approach)? 

 p. 22: Tables 5 & 6: Would the authors be able to provide information (average and SD/range) on the % of total costs per study for the various cost centers? This can be a useful metric in understanding relative resource use across programs, particularly when considering % of costs for personnel and therapeutic foods.

 p. 22: Regarding the need for more evidence on costs of integrating CMAM into primary healthcare, I would add that it would be particularly useful to generate cost estimates from government-delivered programs. The available evidence (which includes references 64 and 65 by Rogers *et al* in Pakistan and Mali) includes substantial support from international non-governmental organizations and therefore likely represents a higher cost than that of a fully integrated program.

 The authors mention that due to several methodological aspects of the included studies, a meta-analysis was inappropriate, and I would agree. Do the authors have any specific recommendations to improve cost estimates for future studies (i.e. more transparency in reporting or a standard set of cost categories to include)? Could the authors perhaps expound on the specific difficulties of comparing or standardizing costs and cost structures across settings, based on their experience in reading and comparing the reviewed analyses? 

 It is appropriate that the GHCC guidelines were used to address quality of evidence. Given that this data was collected and an analysis conducted around trends and gaps in study quality, do the authors have any insights they can share in the discussion as to the most common principles that studies did not adhere to and why that might be the case? This could be useful information to inform and improve future cost analyses in nutrition.

Are the rationale for, and objectives of, the Systematic Review clearly stated?

Partly

Is the statistical analysis and its interpretation appropriate?

Not applicable

Are sufficient details of the methods and analysis provided to allow replication by others?

Yes

Are the conclusions drawn adequately supported by the results presented in the review?

Yes

Reviewer Expertise:

Economic analysis of nutrition interventions

I confirm that I have read this submission and believe that I have an appropriate level of expertise to confirm that it is of an acceptable scientific standard, however I have significant reservations, as outlined above.

## References

[1] : Big Numbers about Small Children: Estimating the Economic Benefits of Addressing Undernutrition. *World Bank Res Obs*.32(1) : 10.1093/wbro/lkw003 107-125 10.1093/wbro/lkw003 28845075PMC5568100

# Response

**Published:** 2020-09-11

**Authors:** Rebecca Njuguna

**Affiliations:** KEMRI-Wellcome Trust Research Organisation, Kenya

**Authors Response**

****

**Reviewer 2: Chloe Puett**

 1. This manuscript presents a systematic review of the under-researched area of economic analysis of nutrition interventions in LMICs, addressing an important gap in the scientific literature. The authors give special attention to costs incurred by households during treatment, which is an often-ignored aspect of economic analysis for nutrition with real implications for intervention coverage, adherence and, ultimately, effectiveness. The review appears to have been well-conducted and the analytical approach is described in detail. However, there are a few points of clarification needed that would help position this article's contributions more specifically relative to the evidence that it presents.

**Thank you for taking your time to review our work and for the helpful comments and suggestions that will help improve our article.**

 2. The objective of the analysis in the introduction is stated as "[determining] the current state of knowledge on costs of child undernutrition treatment to households, health providers, organizations and governments in low and middle-income countries (LMICs)." In the methods section, the inclusion/exclusion criteria section simply states that articles were included that "involved undernutrition or interventions related to undernutrition" and that articles were included that "reported global cost estimates of child undernutrition *treatment*", and goes on later to specify that this focused on anthropometry outcomes and excluded micronutrient deficiencies. The exact inclusion criteria (and preferably the justification for this focus) should be clarified in the introduction. As part of this, the authors should specify what is meant by "treatment" and clearly describe what interventions were/were not under consideration, again with justification if possible. It would be interesting and informative, for example, to know why the authors did not include costs of prevention in the search. 

**Thank you for this comment.**

**We specifically aimed to examine treatment. We considered that prevention, including screening and treating micronutrient deficiencies are themselves broad topics worthy of their own reviews with different considerations in terms of costs and cost-effectiveness.**

**This has been clarified in the introduction section to read:**

**“We only included studies that assessed the cost of treatment interventions (for children with anthropometrically defined wasting or kwashiorkor).  Interventions ranging from supplementary feeding for children with moderate acute malnutrition and therapeutic feeding and other treatments for children with severe acute malnutrition, including during community-based management of severe acute malnutrition (CMAM) as well as facility-based outpatient and inpatient treatment. We excluded prevention interventions, screening and treating micronutrient deficiencies as they are broader topics worthy of their own reviews”**

 3. Given that the introduction refers to stunting outcomes, one wonders why the keywords for stunting (and underweight) and related terms (height-for-age (HAZ), weight-for-age (WAZ), stuntedness, etc) were not included in the search terms listed in Table 1. The included terms would seem to position the paper to be more of a review on economic analysis of acute malnutrition than undernutrition more generally, including chronic undernutrition. 

**Thank you for this comment.**

**Having broader terms such as undernutrition and malnutrition which encompass stunting and underweight, our search term was also able to capture many studies assessing stunting interventions which were included in the review.**

 3. Perhaps due to this oversight in search terms (if my understanding is correct), at least one potentially relevant study does not appear to be included in the review:

 Alderman H *et al*. (2017). Big numbers about small children: Estimating the economic benefits of addressing undernutrition. *The World Bank Research Observer*, 32(1), 107-125 ^1^. 

**Thank you for this comment.**

**This paper appeared in the search but was excluded as it was primarily methodological which was outside the scope of our review.**

 4. p. 18: For the approaches to costing, it should be clarified which of these approaches use institutional accounting data (instead of using unit costs and quantities alone via an 'ingredients' approach). 

**Thank you for this comment.**

**This is included in the Results: Type of economic evaluation and analytical approach section in the second paragraph and in figure 4.**

 5.  p. 18: In addition to the point above, I would recommend that in the sub-section "economic evaluation by perspective" that the authors first define and describe each perspective analyzed and presented. For example it is currently unclear what is the difference between the health providers and institution/program perspective. And when the authors describe the "community volunteer perspective" are they referring to studies which include direct and indirect costs incurred by community volunteers during implementation (which would seem to be more an aspect of a general societal perspective), or is this a broader analytical perspective?  

**Thank you for this comment.**

**We have clarified the definitions in the Results: Economic evaluation by perspective section in article.**

 6. p. 18: Puett *et al* 2013 also considers costs for CMAM delivered by community volunteers, and includes the indirect costs of their time allocation in the analysis 

**Thank you for this comment.**

**This has been added in the article in the “Results: Community volunteers perspective” subsection to read:**

**In addition, a paper done in Bangladesh assessing the cost-effectiveness of CMAM delivered by CHWs found out that this was more cost-effective (US$26 per DALY averted)than inpatient treatment (US$1344 per DALY averted). Each CHWs was paid a monthly stipend of US$11.80 during this study.**

 7.  pp 20-21: Would it be possible for the authors to break down costs of CMAM by programs implementing traditional facility-based CMAM versus programs delivered by community volunteers (i.e. a community case management approach)?  

**Thank you for this comment.**

**This has been added in the “CMAM” section to read:**

**“On average cost per child for the CMAM programs implemented by community volunteers was $216 while CMAM implemented in traditional facility-based programs was $300 per child”**

 8. p. 22: Tables 5 & 6: Would the authors be able to provide information (average and SD/range) on the % of total costs per study for the various cost centres? This can be a useful metric in understanding relative resource use across programs, particularly when considering % of costs for personnel and therapeutic foods. 

**Thank you for this comment.**

**This has been added in both table 5 & 6**

**In addition, the data extraction excel sheet containing the cost data per study has been shared in the underlying data section in the article.**

 9. p. 22: Regarding the need for more evidence on costs of integrating CMAM into primary healthcare, I would add that it would be particularly useful to generate cost estimates from government-delivered programs. The available evidence (which includes references 64 and 65 by Rogers *et al* in Pakistan and Mali) includes substantial support from international non-governmental organizations and therefore likely represents a higher cost than that of a fully integrated program.

**Thank you for this comment and addition.**

**We agree that understanding costs in integrated government-delivered programmes is key.**

**This has been added in the conclusion section to read:**

**“Additionally, current cost estimates on integrated programs include substantial support from international organisations which may represent higher costs. Therefore, there is need for more studies to generate cost estimates of integrated programs from government delivered programs to represent the actual situation”.**

 10. The authors mention that due to several methodological aspects of the included studies, a meta-analysis was inappropriate, and I would agree. Do the authors have any specific recommendations to improve cost estimates for future studies (i.e. more transparency in reporting or a standard set of cost categories to include)? **Could the authors perhaps expound on the specific difficulties of comparing or standardizing costs and cost structures** across settings, based on their experience in reading and comparing the reviewed analyses?  

**Thank you for this comment.**

**This has been added in the article in the “Limitations” section to read:**

**“A limitation inherent in the available data was that there was a wide range of cost outcomes and unit measurements for some of the outcomes, cost categories for similar cost centres varied a lot among the studies. Thus,** **meta-analysis was inappropriate”.**

 11. It is appropriate that the GHCC guidelines were used to address quality of evidence. Given that this data was collected, and an analysis conducted around trends and gaps in study quality, do the authors have any insights they can share in the discussion as to the most common principles that studies did not adhere to and why that might be the case? This could be useful information to inform and improve future cost analyses in nutrition.

**Thank you for this comment.**

**This has been added in the article in section “Limitations” to read:**

**“For instance, most articles did not mention the perspective, costing approach used and did not conduct sensitivity analysis to characterise uncertainties in the reported costs outcomes”.**

**This has also been added in the “conclusion” section to read;**

**“We also recommend that for easy and comprehensive secondary analysis all items as listed in the GHCC guidelines including explicitly stating the perspective of the analysis, costing methods used, conducting sensitivity analysis should be adhered to by authors”.**

# Peer review: Reviewer response for version 1

**DOI:** 10.21956/wellcomeopenres.17307.r38356

**Published:** 2020-04-15

**Authors:** Max Oscar Bachmann

**Affiliations:** 1 Norwich Medical School, Faculty of Medicine and Health Sciences, University of East Anglia, Norwich, UK

This is an excellent systematic review of evidence about an important subject that will be of value to a wide range of readers and organisations involved in under nutrition in low and middle income countries. The methods are appropriate and clearly set out. The results are clearly presented. One of the main findings is the variety of methods and heterogeneity of results, which make it inappropriate to pool and summarise the results quantitatively, as the article points out. However, as the authors discuss too, some general findings are apparent, especially the lower cost of CMAM compared to hospital inpatient care, and the importance of personnel costs.

 A possible limitation of the article is that there is little methodological discussion about which of the diverse methods reviewed provide the most robust and useful results, and what methods would should be best for future research. However, as the aim of the study was not methodological but was simply to review existing evidence, that is understandable and acceptable. 

 I have only a few suggestions for minor amendments:

- Abstract, Background. Change “….knowledge on costs of child undernutrition” to “….knowledge on costs and cost-effectiveness of child undernutrition” (to match the title).
- Abstract, Results: Consider adding a sentence or two about cost-effectiveness, such as range of costs per life saved and per DALY gained, because these are important for decisions about resource allocation and priorities.
- Methods, Statistical Analysis: The sentence beginning “We also assessed the main cost drivers…” implies that statistical analysis was used to identify the main cost drivers and coping strategies, which left me wondering what kinds of analysis that was. The results (Tables 5 and 6) show that this simply entailed reporting the mean (SD) and median costs reported for each type of cost. I suggest editing that sentence to make the descriptive method clearer, as in the preceding sentence.
- Results, page 16, Studies by region and continent: “… more than 75% of countries…”. Should that be “… more than 75% of studies were in countries…”, because in Figure 2 the unit of analysis is articles, not countries?
- Table 3, study 2 (Bachmann). Cost per death averted was USD1760; cost per life year saved was not reported (this was correctly reported in Table 2).

Are the rationale for, and objectives of, the Systematic Review clearly stated?

Yes

Is the statistical analysis and its interpretation appropriate?

Yes

Are sufficient details of the methods and analysis provided to allow replication by others?

Yes

Are the conclusions drawn adequately supported by the results presented in the review?

Yes

Reviewer Expertise:

NA

I confirm that I have read this submission and believe that I have an appropriate level of expertise to confirm that it is of an acceptable scientific standard.

# Response

**Published:** 2020-09-11

**Authors:** Rebecca Njuguna

**Affiliations:** KEMRI-Wellcome Trust Research Organisation, Kenya

**Cost and cost-effectiveness analysis of treatment for child undernutrition in low- and middle-income countries: A systematic review [version 1; peer review: 1 approved, 1 approved with reservations]**

**Authors response**

**#Reviewer 1: Max Oscar Bachmann: Approved**

 1. This is an excellent systematic review of evidence about an important subject that will be of value to a wide range of readers and organisations involved in under nutrition in low and middle-income countries. The methods are appropriate and clearly set out. The results are clearly presented. One of the main findings is the variety of methods and heterogeneity of results, which make it inappropriate to pool and summarise the results quantitatively, as the article points out. However, as the authors discuss too, some general findings are apparent, especially the lower cost of CMAM compared to hospital inpatient care, and the importance of personnel costs.

**Thank you for taking your time to review our work and for the helpful comments and suggestions that will help improve our article.**

 2. A possible limitation of the article is that there is little methodological discussion about which of the diverse methods reviewed provide the most robust and useful results, and what methods would should be best for future research. However, as the aim of the study was not methodological but was simply to review existing evidence, that is understandable and acceptable.  

**Thank you for this comment.**

**We used the Global Health Cost Consortium guidelines to assess the quality of the articles included and noted the heterogeneity of costing methods used. This is mentioned on the methods section “Quality assessment of studies”. The results according to the assessment by the GHCC guidelines are on Table 7.**

**However, we did not assess and analyse the articles’ diverse methods as this was outside the scope of our study.**

 3. Abstract, Background. Change “….knowledge on costs of child undernutrition” to “….knowledge on costs and cost-effectiveness of child undernutrition” (to match the title).

**Thank you for this comment. This has been changed in the article (Abstract: Background) to read:**

**“The aim of this study was to determine the current state of knowledge on costs and cost-effectiveness of child undernutrition treatment to households, health providers, organizations and governments in low and middle-income countries (LMICs)”.**

 4. Abstract, Results: Consider adding a sentence or two about cost-effectiveness, such as range of costs per life saved and per DALY gained, because these are important for decisions about resource allocation and priorities.

**Thank you for this comment. This has been changed in the article (Abstract: results) to read:**

**We also assessed the cost effectiveness of community-based management of malnutrition programs (CMAM). Cost per disability adjusted life year (DALY) averted for a CMAM program integrated into existing health services in Malawi was $42. Overall, cost per DALY averted for CMAM ranged between US$26 and US$53, which was much lower than facility-based management (US$1344)”**

 5. Methods, Statistical Analysis: The sentence beginning “We also assessed the main cost drivers…” implies that statistical analysis was used to identify the main cost drivers and coping strategies, which left me wondering what kinds of analysis that was. The results (Tables 5 and 6) show that this simply entailed reporting the mean (SD) and median costs reported for each type of cost. I suggest editing that sentence to make the descriptive method clearer, as in the preceding sentence. 

**Thank you for this comment. This has been changed in the article in the Methods:Statistical analysis section to read:**

**“We reported the means, medians and ranges of the direct and indirect costs according to the perspectives adopted by the included studies. The mean and median costs reported were used to identify the main cost drivers for each perspective. We also reviewed coping strategies reported by the included articles.”**

 6. Results, page 16, Studies by region and continent: “… more than 75% of countries…”. Should that be “… more than 75% of studies were in countries…”, because in Figure 2 the unit of analysis is articles, not countries? 

**Thank you for this comment.**

**This has been changed in the article in the Results section:Studies by region and continent to read;**

**“With reference to the World Bank classification of countries, more than 75% of these studies were conducted in either low income or lower middle economies (with GNI per capita of less than $3,996).”**

 7. Table 3, study 2 (Bachmann). Cost per death averted was USD1760; cost per life year saved was not reported (this was correctly reported in Table 2).

**Thank you for this comment.**

**“Table 2 reports : Mean cost per child was $203 CTC cost, $53 per DALY gained and $1760 per life saved.**

**Table 3 reports: $53 per DALY gained, $1760 per life year saved.”**
